# Supplementary material for: Micro and macroevolution of sea anemone venom phenotype
Source: Nat Commun. 2023 Jan 16;14:249. doi: 10.1038/s41467-023-35794-9 (PMC9842752; doi:10.1038/s41467-023-35794-9)
Supplement: Supplementary file 1 — Supplementary Information [file 41467_2023_35794_MOESM1_ESM.pdf]

Micro and macroevolution of sea anemone venom phenotype

Supplementary Information

Supplementary Figures

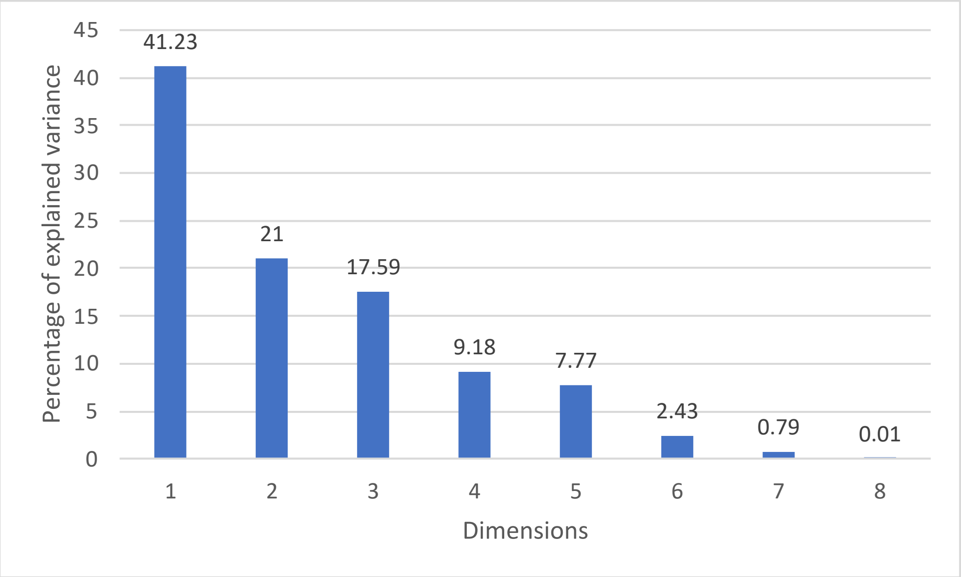

Supplementary Figure 1. Scree plot of the phylogenetic covariance analysis of toxin expression

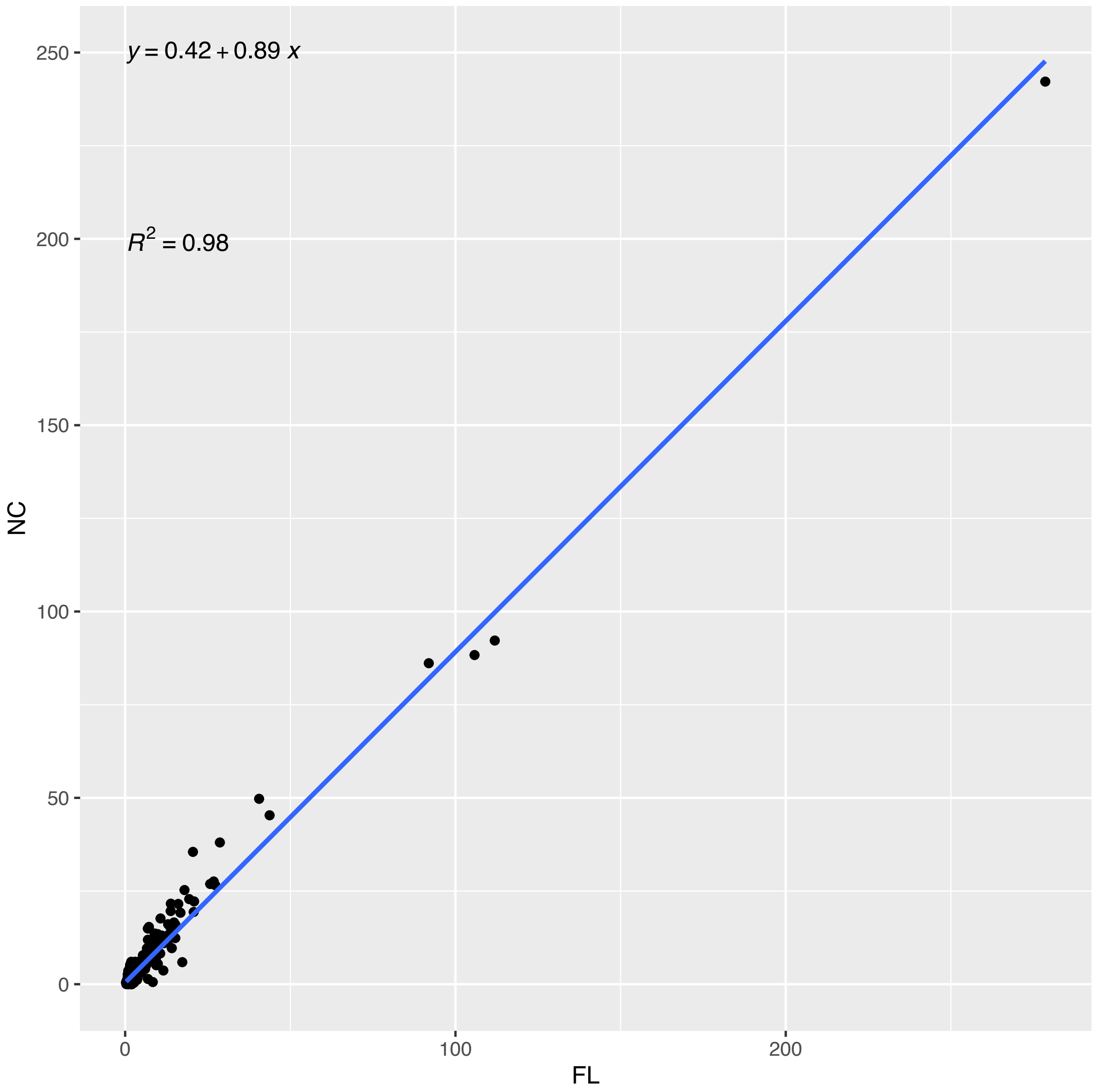

Supplementary Figure 2. Linear regression comparing proteomes average label-free quantification (LFQ) per million from Florida and North Carolina, on the x- and y-axis, respectively. Data used can be found in Supplementary table 17.

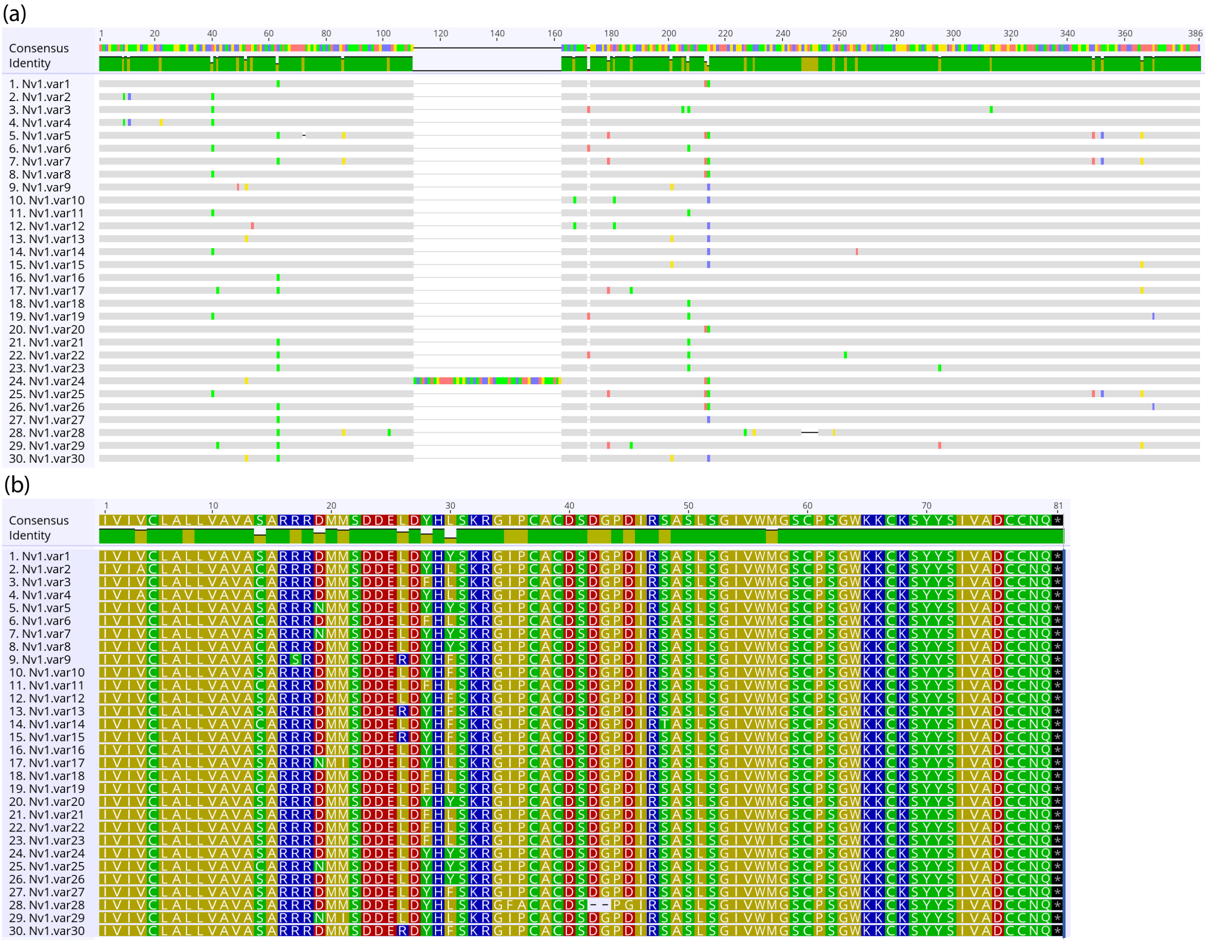

Supplementary Figure 3. Multiple sequence alignment of nucleotides (a) and amino acids (b) from all Nv1 paralogs identified in the amplicon analysis.

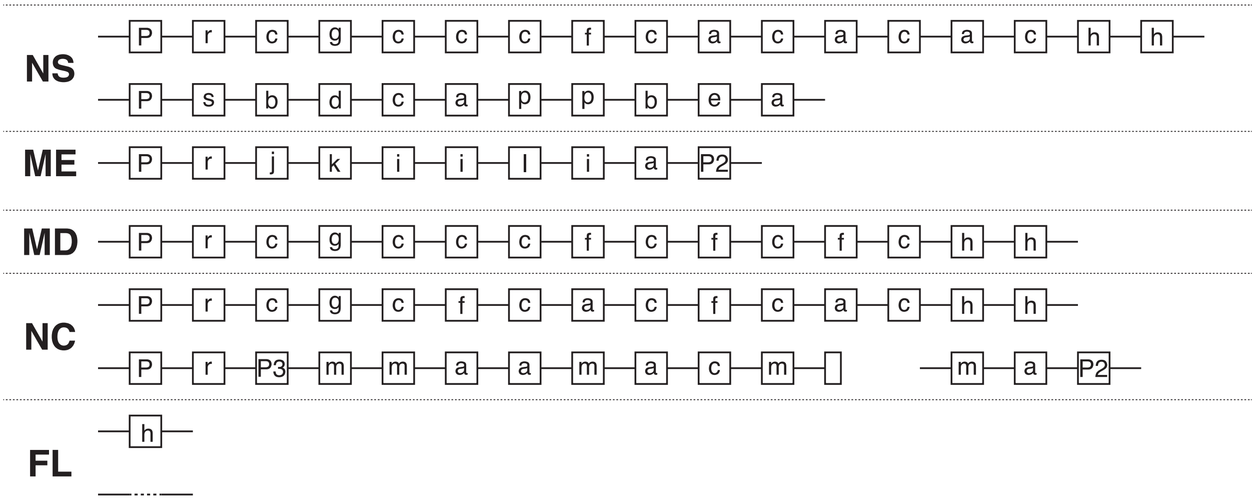

Supplementary Figure 4. Genomic arrangement of *Nv1* amino acid variants at the *Nv1* locus across individuals. Each letter code represents a distinct variant. Shared pseudogenes are labelled (P, P2 and P3).

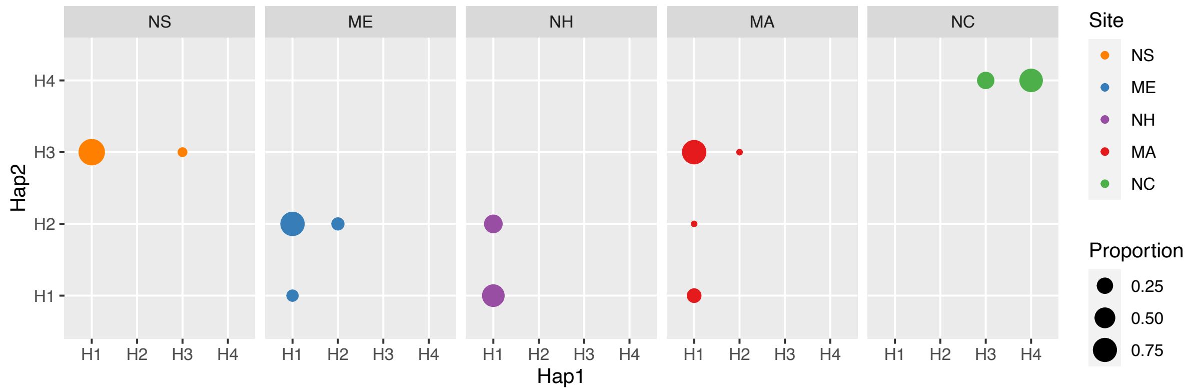

Supplementary Figure 5. Relative *Nv1* locus genotype frequencies between sampling sites.

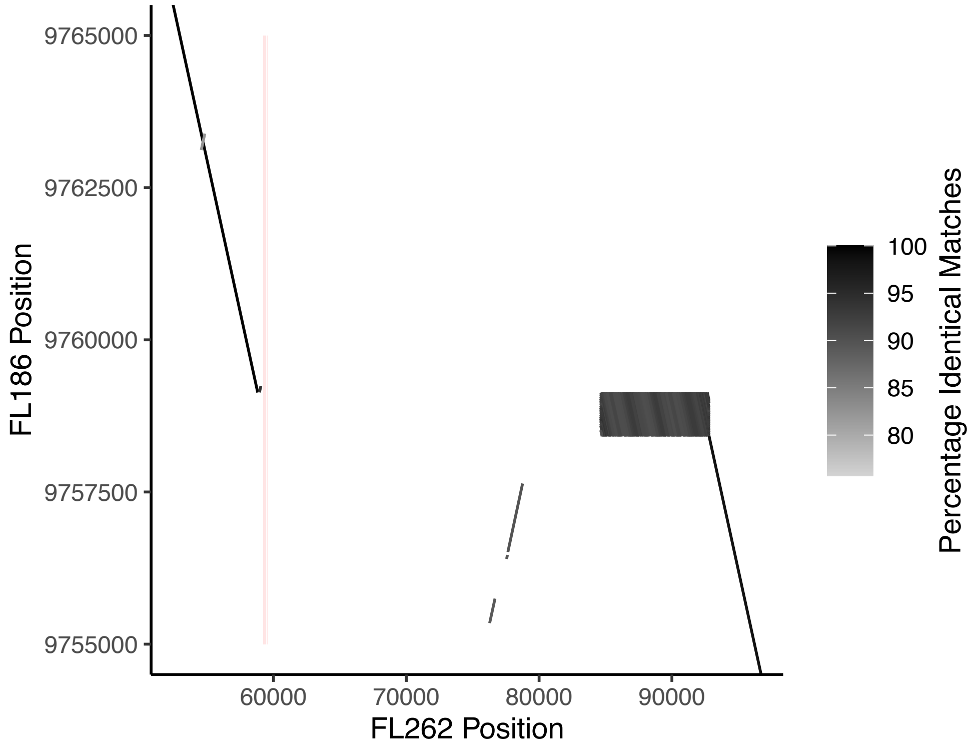

Supplementary Figure 6. Dotplot of BLASTn results between the two Florida haplotypes. The red bars indicate the location of the *Nv1* exons in haplotype FL262.

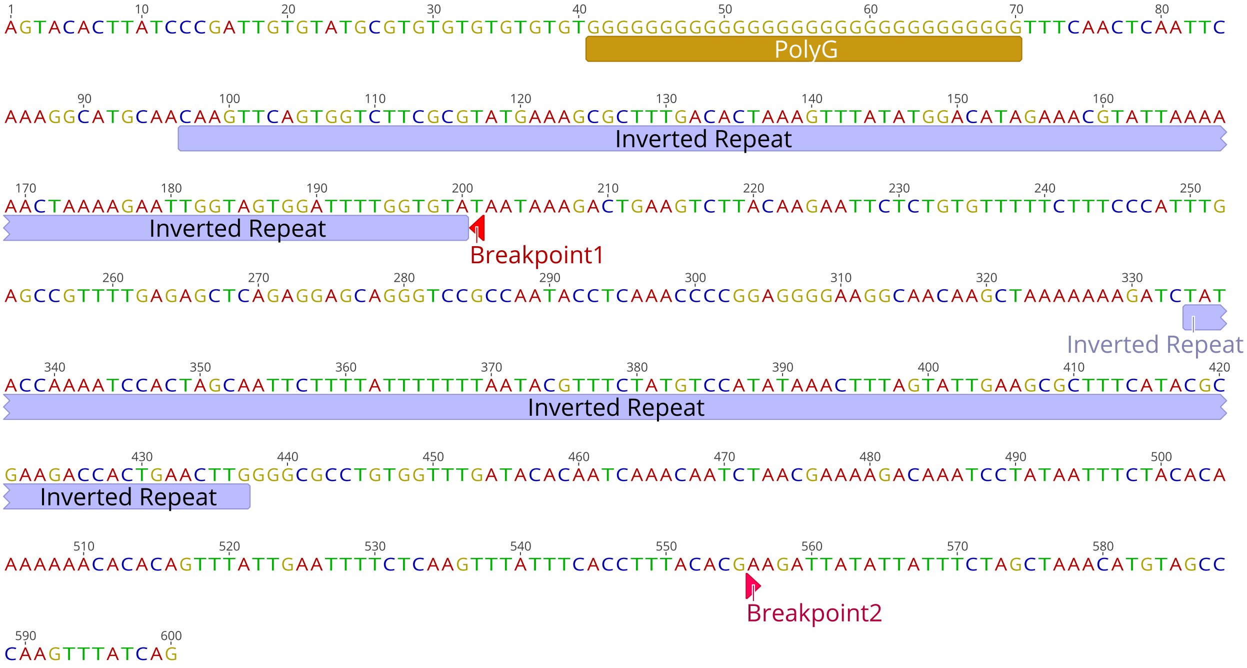

Supplementary Figure 7. Annotation of genomic locus (NS.tig889:951254-951854) associated with deletion breakpoints. Sequence features associated with non-b DNA structures are annotated in gold and blue. Breakpoints 1 and 2 refer to the breakpoints associated with the Nv1 locus deletion in the zero-copy haplotype, and the pseudogenes associated with NC.H4 and ME.H2, respectively.

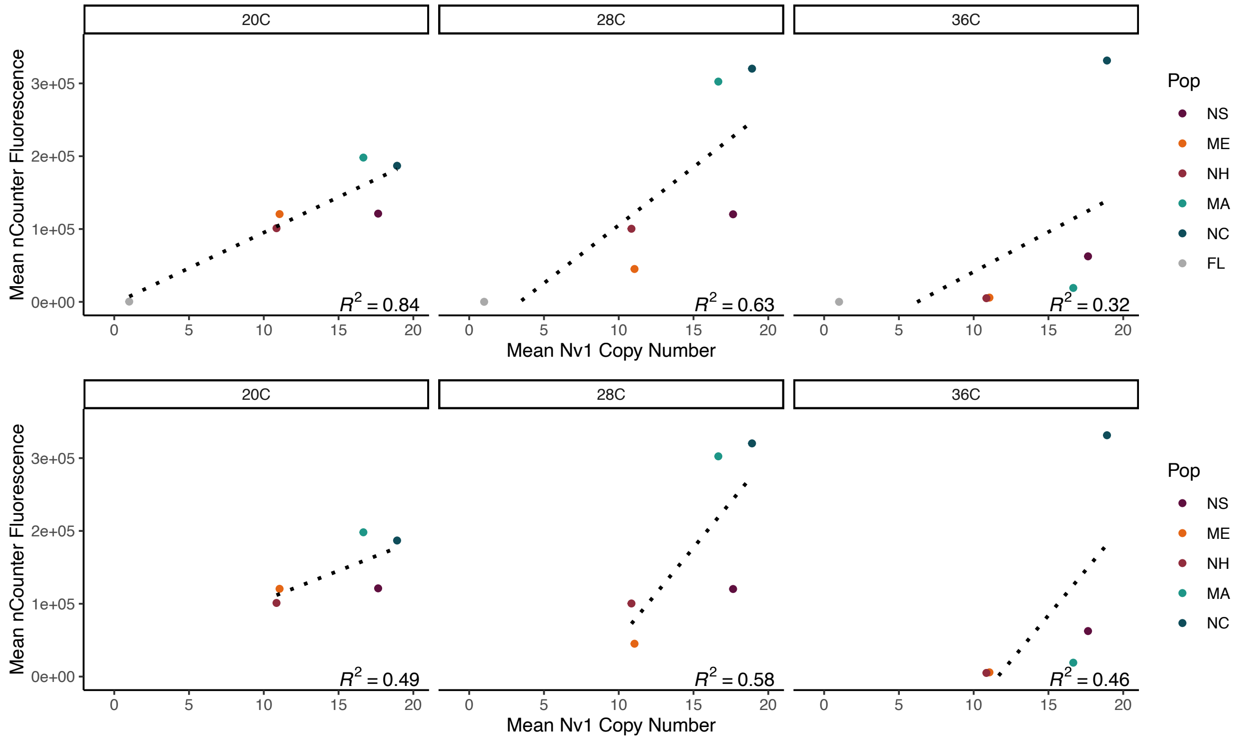

Supplementary Figure 8. Nv1 copy number vs transcript abundance at three temperatures (20°C, 28°C, and 36°C) across *N. vectensis* populations with (above) and without (below) the Florida population.

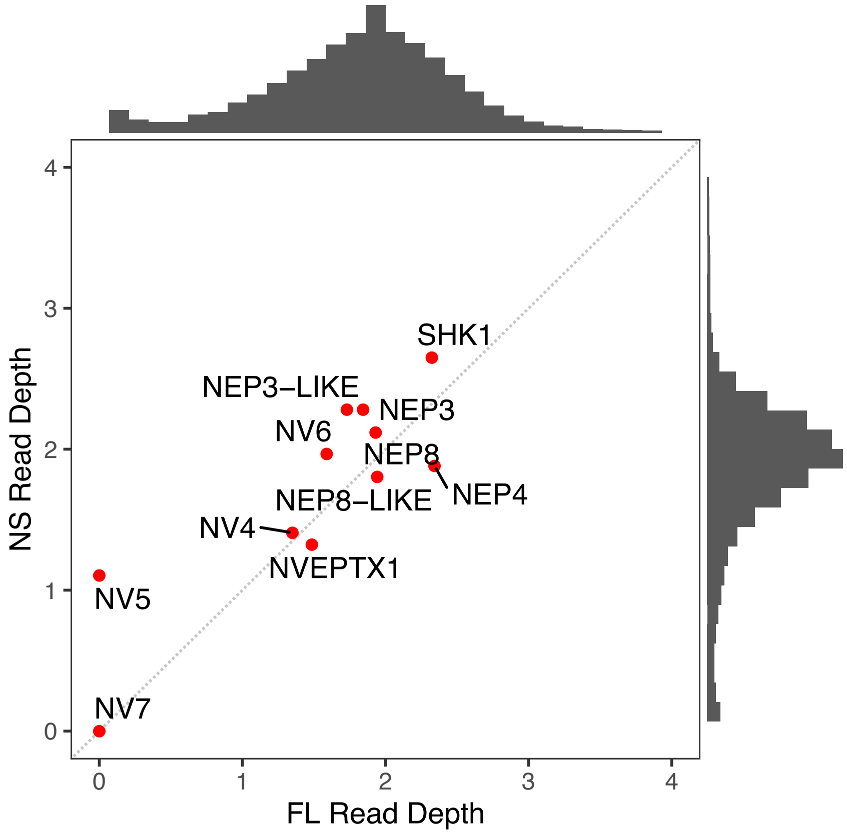

Supplementary Figure 9. Modal read depth for Nova Scotia and Florida individuals at 11 other *N. vectensis* toxin gene loci. Read depths are normalized to diploid copy number. Histograms show a representative read depth distribution sampled across 1 million bases of the *N. vectensis* genome.

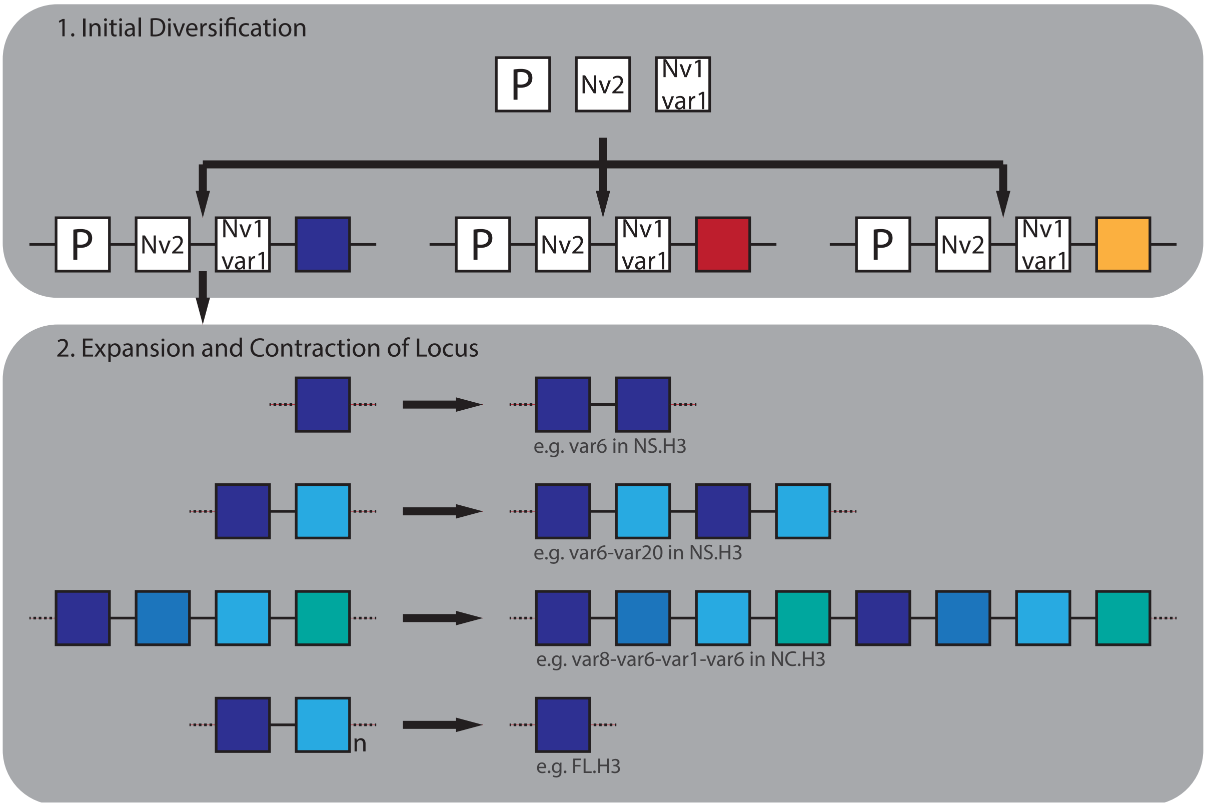

Supplementary Figure 10. Schematic representation of Nv1 locus evolution. 1) Ancestral locus (containing the pseudogene, *Nv2*, and *Nv1.var1*) undergoes initial duplication and diversification events. 2) The resulting loci subsequently expand through replication slippage generating duplicated blocks of single or multiple genes. These loci are also subject to contraction events that appear to be mediated by deletions associated with non-b DNA structures.
