# Supplementary material for: Micro and macroevolution of sea anemone venom phenotype
Source: Nat Commun. 2023 Jan 16;14:249. doi: 10.1038/s41467-023-35794-9 (PMC9842752; doi:10.1038/s41467-023-35794-9)
Supplement: Supplementary file 3 — Description of Additional Supplementary Files [file 41467_2023_35794_MOESM3_ESM.pdf]

Description of Additional Supplementary Files

Supplementary Data 1. Sea anemone comparative transcriptomics and completeness.

Supplementary Data 2. Toxin expression matrix among sea anemones. Act = Actinioidea, Met = Metridioidea, Edw = Edwardsioidea

Supplementary Data 3. Phylogenetic covariance analysis of toxin expression among sea anemones. NS = Not significant.

Supplementary Data 4. Phylogenetic signal and model of evolution on toxin expression in sea anemones. Brownian motion (BM), Ornstein–Uhlenbeck (OU), or early burst (EB) models

Supplementary Data 5. Phylogenetic covariance analysis and fitting tissue type as a fixed effect, showing that the confidence intervals overlap zero.

Supplementary Data 6. Toxin copy number in sea anemone genomes identified using BLASTp with KTx3 and NaTx highlighted in cyan as those identified manually.

Supplementary Data 7. Macrosynteny of the number of single-copy orthologs (SCO) shared among sea anemones genomes.

Supplementary Data 8. NEP3 and NEP6 genome loci in sea anemones genomes

Supplementary Data 9. Percent identity for NaTx and KTx3 genes within sea anemones genomes.

Supplementary Data 10. Population transcriptomics for *Nematostella vectensis* representing sequencing depth and completeness.

Supplementary Data 11. Gene expression of *Nv1* and other toxins in *Nematostella vectensis* from different populations. A) A matrix of known toxin genes mapped to reference *Nematostella vectensis* from different populations B) nCounter normalized data of *Nv1* expression and housekeeping genes from different populations at different temperatures.

Supplementary Data 12. Raw quantification cycle values and estimated *Nv1* copy number for *N. vectensis* individuals from five populations along the Atlantic Coast of North America.

Supplementary Data 13. Results of two-way ANOVA (one-tailed test) testing the effect of population and plate on estimates of diploid *Nv1* copy number

Supplementary Data 14. Post hoc test results for *Nv1* diploid copy number across populations. Absolute difference in mean copy number is shown above the diagonal and the p-value from the Tukey-Kramer test controlling for family-wise error rate (a = 0.05) below the diagonal

Supplementary Data 15. Semi-quantitative proteomic results from two Florida and North Carolina populations of *Nematostella vectensis.*

Supplementary Data 16. Perseus statistical analysis performed using semi-quantitative proteomic results from Florida and North Carolina populations of *Nematostella vectensis.*

Supplementary Data 17. Raw data used for linear regression analysis comparing the semi-quantitative proteomic results from two Florida and North Carolina populations of *Nematostella vectensis.*

Supplementary Data 18. Read information for reads spanning *Nv1* haplotypes.

Supplementary Data 19. Transposable element annotations generated by EDTA spanning the *Nv1* locus and 20kb up- and downstream for the Nova Scotia, Florida, and Maryland assemblies.
