# Supplementary material for: Micro and macroevolution of sea anemone venom phenotype
Source: Nat Commun. 2023 Jan 16;14:249. doi: 10.1038/s41467-023-35794-9 (PMC9842752; doi:10.1038/s41467-023-35794-9)
Supplement: Supplementary file 5 — Reporting Summary [file 41467_2023_35794_MOESM5_ESM.pdf]

nature portfolio

Corresponding author(s): Joachim Surm, Edward Smith

Last updated by author(s): Dec 4, 2022

Reporting Summary

Nature Portfolio wishes to improve the reproducibility of the work that we publish. This form provides structure for consistency and transparency in reporting. For further information on Nature Portfolio policies, see our Editorial Policies and the Editorial Policy Checklist.

Statistics

For all statistical analyses, confirm that the following items are present in the figure legend, table legend, main text, or Methods section.

n/a	Confirmed	
☐	☒	The exact sample size (*n*) for each experimental group/condition, given as a discrete number and unit of measurement
☐	☒	A statement on whether measurements were taken from distinct samples or whether the same sample was measured repeatedly
☐	☒	The statistical test(s) used AND whether they are one- or two-sided<br>*Only common tests should be described solely by name; describe more complex techniques in the Methods section.*
☐	☒	A description of all covariates tested
☐	☒	A description of any assumptions or corrections, such as tests of normality and adjustment for multiple comparisons
☐	☒	A full description of the statistical parameters including central tendency (e.g. means) or other basic estimates (e.g. regression coefficient) AND variation (e.g. standard deviation) or associated estimates of uncertainty (e.g. confidence intervals)
☐	☒	For null hypothesis testing, the test statistic (e.g. *F*, *t*, *r*) with confidence intervals, effect sizes, degrees of freedom and *P* value noted<br>*Give P values as exact values whenever suitable.*
☐	☒	For Bayesian analysis, information on the choice of priors and Markov chain Monte Carlo settings
☐	☒	For hierarchical and complex designs, identification of the appropriate level for tests and full reporting of outcomes
☐	☒	Estimates of effect sizes (e.g. Cohen's *d*, Pearson's *r*), indicating how they were calculated

Our web collection on statistics for biologists contains articles on many of the points above.

Software and code

Policy information about availability of computer code

Data collection	New and previously published data
Data analysis	FASTQ-DUMP (2.11.2)<br>Trimmomatic (0.39)<br>Trinity (2.6.6)<br>BUSCO (4)<br>Bowtie2 (2.3.4.3)<br>RSEM (v1.3.1)<br>ORFfinder (0.4.1)<br>CD-HIT (4.8.1)<br>Orthofinder (2.2.7)<br>MAFFT (v7.407)<br>Pal2Nal (v14)<br>IQTREE (1.6.12)<br>https://agneeshbarua.github.io/Many-options-supplementary/<br>MCMCglmm (2.33)<br>SURFACE (0.5)<br>pulsR (1.0)<br>https://agneeshbarua.github.io/LevyModels/<br>Phytools (1.0-3)<br>Splign (Online version: https://www.ncbi.nlm.nih.gov/sutils/splign/splign.cgi)<br>BLAT (37x1)<br>BLAST (2.8.1)

SignalP (v5)
CLANS (29.05.2012)
EMBOSS (6.6.0.0)
MaxQuant (2.0.3.0)
Perseus (1.6.2.2)
Canu (2.0)
Canu (2.1)
Guppy (4.5.2)
gcpp (1.9.0)
bam2fasta (1.3.1)
pbmm2 (1.3.0)
samtools (1.9)
bamtools (2.5.1)
https://gist.github.com/wdecoster/1ab9adac7c8095498ff91ee22468eaac#file-nanopore_timefilt-py
minimap2 (2.17)
seqkit (0.16.1)
racon (1.4.21)
mothur (1.44.3)
cutadapt (2.6)
R (4.0.2)
MEGAX (10.1.8)
BLAST (2.10.1)
EDTA (1.9.6)
https://github.com/edomics/Nv1omics

For manuscripts utilizing custom algorithms or software that are central to the research but not yet described in published literature, software must be made available to editors and reviewers. We strongly encourage code deposition in a community repository (e.g. GitHub). See the Nature Portfolio guidelines for submitting code & software for further information.

Data

Policy information about availability of data

All manuscripts must include a data availability statement. This statement should provide the following information, where applicable:

- Accession codes, unique identifiers, or web links for publicly available datasets
- A description of any restrictions on data availability
- For clinical datasets or third party data, please ensure that the statement adheres to our policy

All data used here is publicly available
Previously available data
PRJNA313244
PRJNA317913
PRJNA329297
PRJNA394213
PRJNA507679
PRJNA279590
PRJNA715506
PRJEB21970
PRJNA430035
PRJNA280517
PRJNA213177
PRJEB27893
PRJNA381121
PRJNA464282
https://simrbase.stowers.org/
http://reefgenomics.org/

Data generated
PRJNA831625
PRJNA844989
PRJNA836916
https://doi.org/10.6084/m9.figshare.20115719.v1
proteome exchange (PXD034383).

Field-specific reporting

Please select the one below that is the best fit for your research. If you are not sure, read the appropriate sections before making your selection.

☐ Life sciences ☐ Behavioural & social sciences ☒ Ecological, evolutionary & environmental sciences

For a reference copy of the document with all sections, see nature.com/documents/nr-reporting-summary-flat.pdf

Ecological, evolutionary & environmental sciences study design

All studies must disclose on these points even when the disclosure is negative.

Study description	We analyze publicly available sea anemone transcriptomes and field-collected Nematostella vectensis samples to investigate the molecular processes shaping venom phenotypes across macro- and microevolutionary scales.
Research sample	There were two main groups of research samples used in this study:<br>1. Sea anemone transcriptomes: Publicly available transcriptomes were sampled to represent the breadth of toxin gene composition across sea anemones (see Data section for accessions).<br><br>2. Nematostella vectensis adult individuals. N. vectensis individuals were collected from nine locations (Crescent Beach, Nova Scotia; Saco, Maine; Wallis Sand, New Hampshire; Sippewissett, Massachusetts; Rhode River, Maryland; Ft. Fisher, North Carolina; Georgetown, South Carolina; St. Augustine, Florida) across the Atlantic Coast of the United States and Canada. These sites span the species range along this coastline and were selected based on previous observations and sample availability.
Sampling strategy	Sample sizes for the macroevolutionary analyses of venom phenotype were determined by the availability of sea anemone transcriptomes. These samples span three families and 24 genera. The sampling strategy for the field-collected specimens was designed to enable analysis of toxin gene variability within and between habitats along the Atlantic Coast of North America while accounting for sample availability at these sites. Our previous analyses of genetic relationships of N. vectensis in these locations utilized between 6 and 9 individuals and was a sufficient number to resolve the distribution of genetic variation within and between locations (Reitzel et al. 2013, doi: 10.1111/mec.12228).
Data collection	N. vectensis population transcriptomes: Sequencing libraries were prepared by JM and sequenced on an Illumina HiSeq 4000 at the Duke Center for Genomic and Computational Biology using 150 bp paired-end chemistry.<br>Amplicon sequencing: Amplicon sequencing libraries were prepared by JM and sequenced on an Illumina MiSeq using the MiSeq v3 reagent kit.<br>qPCR: Diploid copy number was estimated using hydrolysis probe-based quantitative PCR on a Applied Biosystems 7500 Fast Real-Time PCR System.<br>Long-read sequencing: Generation of libraries and sequencing on the Pacbio Sequel II and ONT MinION were performed by Brigham Young University DNA Sequencing Center and ES, respectively.<br>Proteomics: MS analysis was performed using a Q Exactive-HF mass spectrometer (Thermo Fisher Scientific, Waltham, MA USA) coupled on-line to a nanoflow UHPLC instrument, Ultimate 3000 Dionex (Thermo Fisher Scientific, Waltham, MA USA) at The Hebrew University of Jerusalem.
Timing and spatial scale	Field samples for the population transcriptomics were collected at a single time point across nine populations spanning the Atlantic Coast of the United States and Canada.<br>Field samples for qPCR and amplicon analyses were collected at five sites across multiple time points to encompass any potential temporal variability. We collected 20 individuals from five locations (Crescent Beach, Nova Scotia; Saco, Maine; Wallis Sand, New Hampshire; Sippewissett, Massachusetts; Ft. Fisher, North Carolina in March 2016, and an additional 10 individuals/month from three of these locations (Saco, Maine; Wallis Sands, New Hampshire; Sippewissett, Massachusetts) in June and September 2016.
Data exclusions	We excluded two samples from the qPCR analyses where the Cq values for either gene were outside of the range used for efficiency estimation and four samples from the amplicon analyses due to insufficient reads (see Methods)
Reproducibility	We did not repeat any experiments for this research. We repeated collections for three locations to discern reproducibility of copy number over time.
Randomization	Individual N. vectensis used for data generation and laboratory culturing from each location were collected haphazardly from each location. These individuals were preserved for extractions without any criteria by the researchers except for the location of origin.
Blinding	We did not use blinding in the generation or analysis of the data. Sequence-based analysis of the replicates of each sample does not involve user-input beyond the sample identification.

Did the study involve field work? ☒ Yes ☐ No

Field work, collection and transport

Field conditions	We did not record the specific field conditions at each sampling site during collection.
Location	Samples were collected from:<br>Location Latitude / Longitude<br>St. Augustine, Florida 29°43'35.1"N 81°15'27.8"W<br>Georgetown, South Carolina 33°19'54.6"N 79°11'59.8"W<br>Ft. Fisher, North Carolina 33°57'33.5"N 77°55'54.8"W<br>Rhode River, Maryland Lab population<br>Sippewissett, Massachusetts 41°35'21.8"N 70°38'16.7"W<br>Wallis Sand, New Hampshire 43°01'42.9"N 70°43'49.9"W<br>Saco, Maine 43°32'33.4"N 70°20'41.9"W<br>Crescent Beach, Nova Scotia 44°13'46.3"N 64°23'06.6"W

Access & import/export	No collection permits were required at our sampling locations.
Disturbance	Samples were collected from habitats known to sustain large N. vectensis populations to minimize any disturbance caused by sampling.

Reporting for specific materials, systems and methods

We require information from authors about some types of materials, experimental systems and methods used in many studies. Here, indicate whether each material, system or method listed is relevant to your study. If you are not sure if a list item applies to your research, read the appropriate section before selecting a response.

Materials & experimental systems

n/a	Involved in the study
☒	☐ Antibodies
☒	☐ Eukaryotic cell lines
☒	☐ Palaeontology and archaeology
☐	☒ Animals and other organisms
☒	☐ Human research participants
☒	☐ Clinical data
☒	☐ Dual use research of concern

Methods

n/a	Involved in the study
☒	☐ ChIP-seq
☒	☐ Flow cytometry
☒	☐ MRI-based neuroimaging

Animals and other organisms

Policy information about studies involving animals; ARRIVE guidelines recommended for reporting animal research

Laboratory animals	Nematostella vectensis (lab strain, adults)
Wild animals	See below
Field-collected samples	Adult N. vectensis were collected from estuaries along the Atlantic coast of the United States and Canada . We collected 20 individuals from five locations (Crescent Beach, Nova Scotia; Saco, Maine; Wallis Sand, New Hampshire; Sippewissett, Massachusetts; Ft. Fisher, North Carolina in March 2016, and an additional 10 individuals/month from three of these locations (Saco, Maine; Wallis Sands, New Hampshire; Sippewissett, Massachusetts) in June and September 2016. Individuals were stored in RNAlater and stored at -20 °C prior to nucleic acid extraction for qPCR and amplicon analyses. At each collection, additional individuals were transported to UNC Charlotte and cultured in the laboratory under standard laboratory conditions (15 parts per thousand artificial seawater, room temperature, fed freshly hatched Artemia 2-3 times per week). In addition, eight adult N. vectensis collected near St. Augustine, Florida were kindly provided by Lukas Schäre (University of Basel). From these laboratory populations, we selected four individual anemones to grow clonal lines for long read sequencing; single individual females from Nova Scotia, Maine, North Carolina, and Florida were grown and bisected to generate the lines.<br><br>Multiple individuals from nine locations in North America were collected. This included Florida, Massachusetts, Maryland, Maine, North Carolina, New Hampshire, New Jersey, Nova Scotia and South Carolina. Individuals from these locations were brought back to the lab and allowed to acclimatize for two weeks.
Ethics oversight	No ethical approval or guidance was required because cnidarians are not regulated by IACUC. protocols managed by the UNC Charlotte Office of Research Protections and Integrity.

Note that full information on the approval of the study protocol must also be provided in the manuscript.
